# Supplementary material for: Chromothripsis during telomere crisis is independent of NHEJ, and consistent with a replicative origin
Source: Genome Res. 2019 May;29(5):737–49. doi: 10.1101/gr.240705.118 (PMC6499312; doi:10.1101/gr.240705.118)
Supplement: Supplemental Material [file supp_gr.240705.118_Supplemental_file_1.zip › contigs/annotated_contigs/DB84/contig.2.DB84_length_351_mean_cov_6.41025641026.docx]

**DB84_length_351_mean_cov_6.41025641026**

```
ATCAGTGACCTGCCTCTTGCCTGTCTGTATGTACAGTATTTAGTCAGAAGGTGGTGCCAAATAGGAGTTCATAGACACAACCTGTCTC|
 >chr16:73561244-73561335 - E=2e-43
AAA|CATGGTTTAAGCTGCTTCTGAGATGTGAAGTTCACAGCCAGCAGTTCTCCTCATCCCCGCTCAGAATTAACCAATAAATTAGAAT
>chr16:73560322-73560585 - E=4e-147
AACTAAGTGAGAAAGGGTGAGACAAAGACAGTGTCAAAACTGGAGGCCAGGGCCTGGGTCATTTTGAATATGAAACGAGGCCTGGTTTA

TGTTTTGTATTTTATAGGTTTTAAAACTAAGCTAAAGTATAACATTAAGGATCAAGGTCAGGGTGTTATCTTTTGTGGGGTGAACA
```
